# Small cell lung cancer with endobronchial growth: A case report

**DOI:** 10.3892/ol.2013.1423

**Published:** 2013-06-25

**Authors:** KOICHI KURISHIMA, KATSUNORI KAGOHASHI, KUNIHIKO MIYAZAKI, TOMOHIRO TAMURA, GEN OHARA, MIO KAWAGUCHI, HIROAKI SATOH

**Affiliations:** Division of Respiratory Medicine, Mito Medical Center, University of Tsukuba, Mito, Ibaraki 310-0015, Japan

**Keywords:** lung cancer, small cell lung cancer, endobronchial growth

## Abstract

The current study presents a rare case of small cell lung cancer (SCLC) with endobronchial growth in a 68-year-old male. Chest CT scans revealed an ill-defined mass in the upper lobe of the right lung, with ipsilateral mediastinial lymph node swelling. An endobronchial polypoid tumor in the right B3 bronchus was located by bronchoscopic examination. The analysis of a biopsy specimen obtained from the tumor resulted in a diagnosis of SCLC. Although extremely rare, this case highlights the importance of considering a diagnosis of SCLC in patients presenting with a pulmonary tumor adjacent to the bronchus, with an endobronchial polypoid lesion.

## Introduction

Although small cell lung cancer (SCLC) is one of the most common histological types of primary lung cancer, endobronchial extension is extremely rare (1,2). A clinical differential diagnosis of endobronchial lesions may include a variety of conditions, such as non-malignant tumors, primary lung carcinoma other than adenocarcinoma and endobronchial metastasis of carcinoma from extrapulmonary organs (3–8). The current study presents the case of a patient with SCLC and endobronchial growth. Written informed consent was obtained from the patient.

## Case report

### Patient presentation

**Figure 1.**
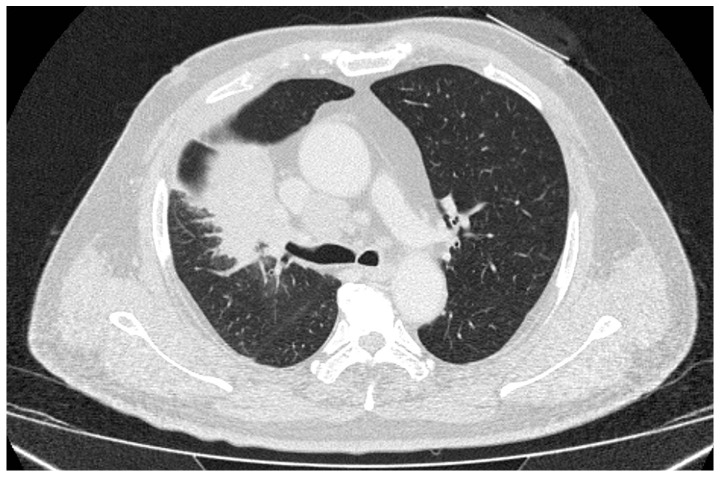
Chest computed tomography (CT) scan revealing an ill-defined mass in the upper lobe of the right lung with ipsilateral mediastinal lymph node swelling.

**Figure 2.**
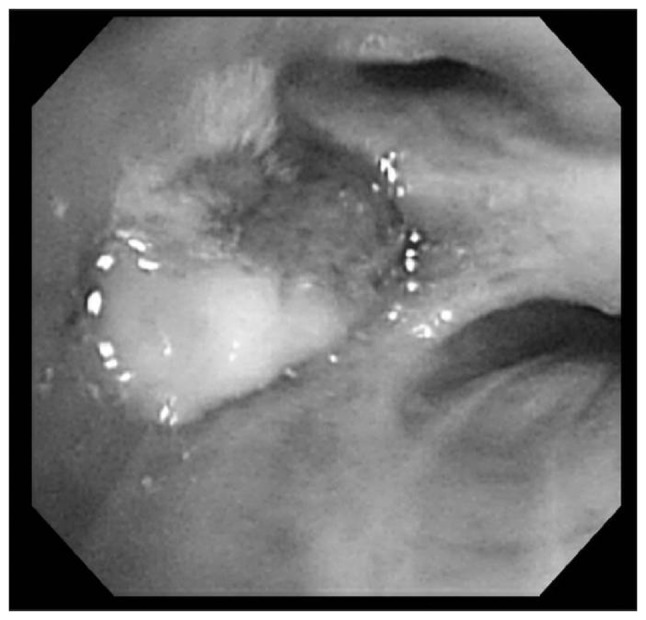
Bronchoscopy revealing a well-circumscribed hypervascular movable tumor located at the right B3 bronchus. The tumor obstructed ∼90% of the lumen and the scope was not able to pass through the narrowed route.

A 68-year-old male was referred to the Mito Medical Center (University of Tsukuba, Mito, Japan) following a three-month history of hoarseness. Upon admission, laboratory tests revealed that the patient had hemoglobin levels of 12.5 g/dl, a hematocrit of 37.2% and a C-reactive protein count of 0.64 mg/dl. The serum levels of neuron-specific enolase were elevated to 156.7 ng/ml. X-ray and computed tomography (CT) scans of the chest revealed an ill-defined mass in the upper lobe of the right lung, with ipsilateral mediastinal lymph node swelling (Fig. 1). A bronchoscopy revealed a well-circumscribed movable tumor located at the right B3 bronchus. The tumor obstructed ∼90% of the lumen and the scope was not able to pass through this region (Fig. 2). Using the bronchoscopy, the whole of the mobile section of the endobronchial tumor was removed easily. Following the removal of the endobronchial tumor, the distal bronchus demonstrated narrowing by a sub-mucosal tumor.

### Pathological analysis

**Figure 3.**
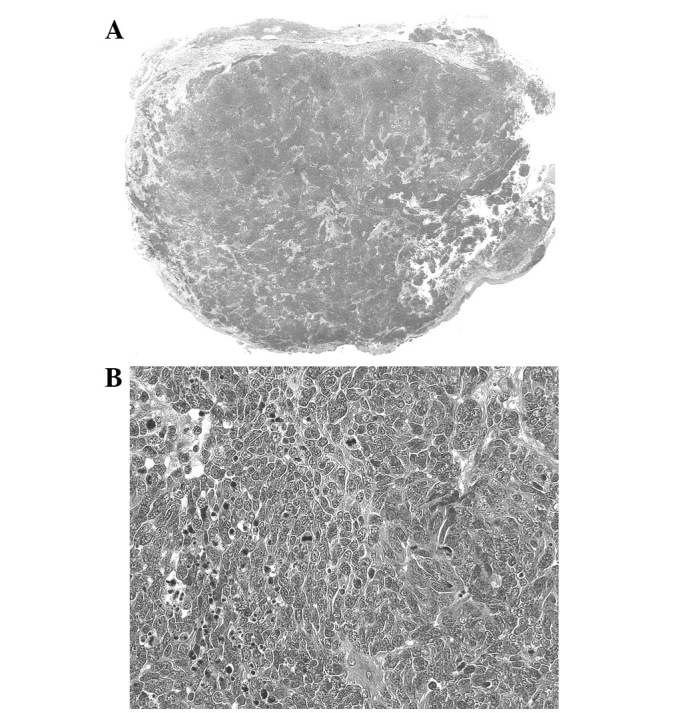
(A) Loupe view of a whole endobraonchial tumor and (B) microscopic features of the endobronchial tumor from the right B3 bronchus.

**Figure 4.**
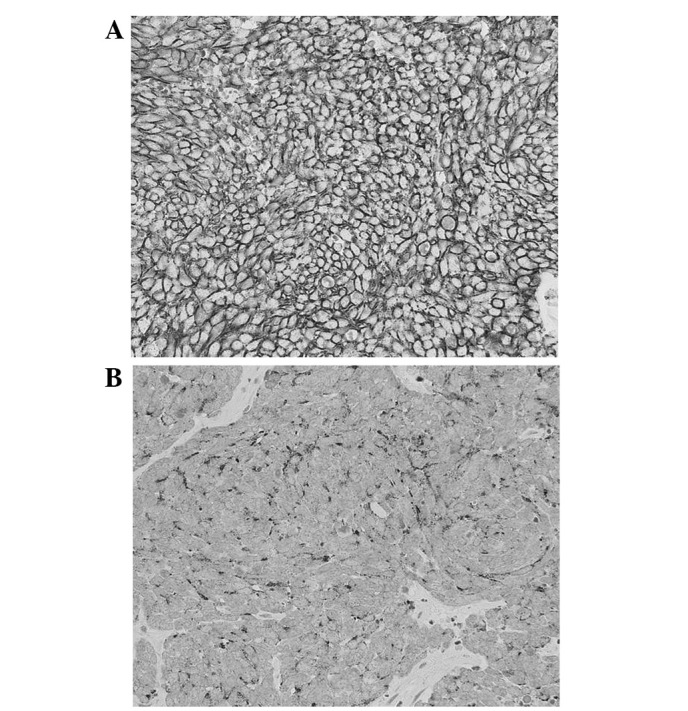
Immunohistochemical analysis revealing positive staining for (A) CD-56 and (B) chromogranin.

The pathological examination of the tumor was consistent with a diagnosis of SCLC (Fig. 3). An immunohistochemical examination revealed positive staining for CD-56 (Fig. 4A) and chromogranin (Fig. 4B).

### Clinical course

Brain magnetic resonance imaging, abdominal ultrasonography and bone scintigraphy scans did not identify a malignancy. The tumor was diagnosed as limited-disease SCLC. The patient received chemoradiotherapy containing four courses of cisplatin (80 mg/m^2^, day 1) and etoposide (100 mg/m^2^, days 1–3), which resulted in a partial response. At six months after the initial therapy, the patient exhibited local recurrence. Despite the second to fourth lines of chemotherapy being less effective, tumor growth at the primary site was not as rapid as previously observed and no distant metastasis was identified. However, 22 months after the initiation of the first course of chemotherapy, the patient succumbed to SCLC.

## Discussion

Radiographical manifestations of endobronchial lesions are considerably variable. Lobar or segmental atelectasis and pneumonic infiltration are commonly observed. The radiological differential diagnosis of an endobronchial mass lesion includes non-malignant tumors, endobronchial metastasis of carcinoma from extrapulmonary organs and primary lung carcinoma (3–8). In addition, the mass-like pulmonary opacity, commonly caused by endobronchial infections, including mucus plugs distal to a centrally obstructing lesion due to a fungus or tuberculosis, also simulate an endobronchial mass on CT scans (9–11). In the current patient, an ill-defined mass in the upper lobe of the right lung was identified, with ipsilateral mediastinal lymph node swelling. Therefore, an endobronchial lesion was not diagnosed in this patient until the bronchoscopy examination.

It is generally accepted that the bronchoscopic findings of endobronchial lesions of primary lung carcinoma are difficult to distinguish from those of metastatic lung carcinoma and non-malignant tumors (12,13). Bronchoscopic examinations are used for the diagnosis of endobronchial lesions as the majority of lesions are within the view and range of the bronchoscopic field. In specific cases, the value of bronchoscopic examinations may be limited as the admixture of necrotic material may prevent the collection of a sufficient specimen for diagnosis (9–11), however, the formation of a pathological diagnosis using specimens obtained by a bronchoscopic biopsy is mandatory for the generation of a correct diagnosis. Non-malignant endobronchial tumors generally have a smooth surface with uniform color (14). The most common sites associated with endobronchial metastasis are the breast, kidney and colon (5–7). Metastatic endobronchial tumors often presents as polypoid or nodular lesions covered with necrotic material (15). Squamous cell carcinoma is the most common histological type of primary lung carcinoma in a central location and with endobronchial extensions (4). In this cell type, polypoid lesions with a rough surface covered with necrotic material are the most predominant lesions (16,17). Although SCLC with endobronchial growth is extremely rare, specific cases have been reported (18,19). Ramaraju *et al* reported an unusual case of centrally-located combined SCLC with a squamous cell component, which was diagnosed by an endo-bronchial lung biopsy (18). In addition, Lee *et al* presented the case of a 51-year-old male with SCLC and an endobronchial mass in the left main bronchus (19). In these cases, the tumors contained elements of SCLC mixed with a component of squamous cell carcinoma (18,19). Patients with combined SCLC have a higher incidence of peripheral lesions, and as a consequence, the central location of the tumor in the present case was unusual. The low incidence rate and often small and inadequate specimens obtained by bronchoscopic biopsy make it difficult to diagnose combined SCLC without surgical resection (20).

In the present case study, the SCLC originated endobronchially from the upper lobe of the lung. Chest CT scans revealed that its location was central. A bronchoscopic examination identified an irregularly covered partly-whitish tissue, representative of the necrotic material coverage. Bronchoscopically, squamous cell lung cancer was considered as a differential diagnosis, although no such component was observed pathologically in the present study. The tumor was not found to exhibit necrotic material or other cell types of lung cancer and an admixture of fibrin in the outer stratum was found, indicative of necrotic material.

Despite SCLC with endobronchial growth being an extremely rare tumor presentation, the clinical evaluation of this diagnosis in patients presenting with a pulmonary tumor adjacent to the bronchus and an endobronchial polypoid lesion must not overlooked.
